# Pyorrhœa Alveolaris

**Published:** 1886-08

**Authors:** Alfred R. Starr

**Affiliations:** New York City


### PYORRHŒA ALVEOLARIS.

Lecture before the Class of the New York College of Dentistry, Session of '85 and '86.

BY ALFRED R. STARR, M. D., D. D. S., NEW YORK CITY.

Pyorrhœa Alveolaris, sometimes called Rigg's disease, catarrhal or suppurative gingivitis or ulitis, and alveolar pyorrhœa, is a disease of which much has been written, but as yet little is known. It is described by some as a disease characterized by a flow of pus from the tooth sockets. The effect upon the gums and alveoli differs very materially from the usual results of salivary calculus, in that in this disease the ulcerative process or retrograde metamorphosis is most marked in the pericementum and alveolus, while the gums are comparatively free. In this affection the destruction and separation of the pericementum and the absorption of the alveoli are greater and more rapid than the recession of the gums, thus resulting in the formation of deep pockets between the gums and the teeth, from which pockets exudes an ichorous or sanious discharge. In cases of salivary calculus proper, with no secondary sanguinary deposit, the recession of the gums, destruction of the pericementum and absorption of the alveolus occur slowly, and the process is limited to the immediate vicinity of the deposit; so that if we go a little beyond the point of contact of the deposit with the gum, we will find the pericementum and alveolus in quite a normal condition.

This is the case, even when the deposit has encroached upon the alveoli almost to the apices of the roots. Even in these cases we may have a fetid, sanious discharge, but instead of proceeding from deep pockets it comes from the tissues in the immediate vicinity of and directly underlying the deposit. If any pockets are formed in these cases of salivary calculus they are very shallow, and the destruction of the pericementum and absorption of the alveoli show little tendency to increase any more rapidly than the ulceration and recession of the gums. In pyorrhœa alveolaris there is frequently no recession of the gums, little or no salivary calculus about the necks of the teeth, and yet we have extensive involvement of the pericementum and alveolus, and usually, if not always, the presence of the dark or sanguinary variety of tartar on the roots.

We sometimes see the manifestations of these two affections, viz., salivary calculus and pyorrhœa alveolaris, on one and the same tooth. Thus, on a lower incisor we may have a large accumulation of salivary calculus on the lingual surface, which seems to almost crowd the gum and alveolus before it, while on the proximal and labial surfaces we may find very little salivary deposit, but in its stead a deposit of sanguinary calculus on the root, extending for some distance under the gum. In the case of salivary calculus proper, the deposit precedes and causes destruction of the pericementum, while in this disease some peculiar irritation of the pericementum precedes and causes calcareous deposit.

The etiology of pyorrhœa alveolaris is very obscure. Authorities are very evenly divided as to whether the causes are constitutional or local. Some regard it as a localization of a systemic debility, while others believe it to be due entirely to local causes, and amenable to local surgical treatment. Some attribute the occurrence of the disease entirely to the presence of tartar and its effects upon the surroundings of the teeth, while others say that while tartar is usually present it is only a concomitant or sequence of the affection, and never the cause. Those who maintain the latter view declare that the disease sometimes occurs without the presence of any tartar. Professor Abbott has said that he has never seen a case of pyorrhœa alveolaris without the presence of some deposit, either calcular, serumal, or sanguineous.

I have met with cases in which I have been unable to detect the presence of incrustations, but I would not venture positively to state that there were none present, for there might easily have been some soft, or even calcareous deposit at some inaccessible point, or possibly the lime salts may have been deposited and accomplished the results, and been subsequently removed. Constitutional dyscrasia (hereditary or acquired), extreme density and low degree of vitality of the teeth, suppression of habitual secretions, catarrhal inflammation, the presence of bacteria, of foreign deposits (salivary, serumal or sanguineous), and local irritation from the use of wedges, ligatures, rubber dam, etc., have been assigned as causes.

The influence of heredity in pyorrhœa is often quite marked, the disease being transmitted through several generations. Cases have been noticed in which children born before the acquisition of the disease by the parent or parents have been exempt, while those born subsequently have developed it at quite an early age. Among the cases due to acquired constitutional predisposition may be cited those caused by mercurialization, or some peculiarity of diet, nutrition, or nervous influence.

Pyorrhœa alveolaris very frequently follows mercurial salivation. Professor Abbott is on record as having said that he believes every person who has been salivated suffers more or less from this trouble. The statement has been made that pyorrhœa alveolaris never occurs except in persons who have been salivated, but this theory has not been generally accepted, and I do not believe it is founded on fact.

It is believed by many that excessive use of chloride of sodium will sometimes cause pyorrhœa alveolaris. In support of this theory it is said that the disease is very common among sailors or laborers who subsist largely on salt meats. Some authors assert that imperfect elimination of urea is its principal constitutional antecedent.

Owing to the fact that the disease occurs most frequently in adult life, and in mouths in which there is little tendency to decay, it has been supposed that the immunity from decay implies extreme density and low degree of vitality in the structure of the teeth, which may result in a final severance between them and the more highly vitalized contiguous parts, and thus constitute a predisposing cause of this disease.

In support of the theory that suppression of habitual secretions may aggravate or incite this affection, Dr. Rehwinkel, in the November number of the Cosmos for 1877, cites the case of a young lady aged eighteen, otherwise healthy, and with no accumulation of salivary calculus, in whom the teeth became very loose, presumably from the fact that the menses had never been established. The extraction of two or three of her teeth, although they were very loose, produced violent and persistent hemorrhage. Local treatment and hygienic measures checked the progress of the malady, and when, after some months, menstruation was established, the disease disappeared and the remaining teeth became firm. In a recent article in the Dental Cosmos (November, 1885), Dr. Patterson has said that he believes the disease to be of a catarrhal nature, and he also inclines to the belief that the calcular deposits are simply the result or sequence of the disease. Dr. Patterson states that in the cases he has observed he has found co-existing nasal, pharyngeal, or laryngeal catarrh (generally combined), in every instance. He believes the disease is generally caused by infection from a preexisting catarrh of the nose or throat, but states, also, that the catarrhal condition of the mouth may originate in that cavity, and not be due to infection, or (I think he should have said) extension of the disease, at all. These primary cases, he thinks, are most apt to occur in those who are in the habit of breathing through the mouth. In support of his theory Dr. Patterson cites the following points of similarity in the pathology of the two diseases, viz:—Nasal catarrh and pyorrhœa alveolaris.

1st. The similar appearance of the affected mucous membrane in both diseases and in the various stages of each.

2d. The identical character of the effusions, viz.: first serous, containing numerous epithelial scales, and then becoming filled with pus and blood corpuscles.

3d. The infectious nature of both diseases, nasal catarrh being contagious and sometimes epidemic, pyorrhœa alveolaris frequently showing a tendency to spread from one tooth to the next, until all may be affected.

4th. The similar burrowing of pus in each trouble.

5th. The tendency in each to destruction of periosteum and underlying bone.

6th. The calcareous deposits occurring in each disease. (Deposits of phosphate and carbonate of lime are sometimes formed in cases of nasal catarrh.)

It is possible that the predisposing or constitutional cause of pyorrhœa alveolaris may, in some instances, be a tendency to catarrhal inflammations; but I do not believe, as does Dr. Patterson, that this disease is transmitted from a pre-existing catarrh of the nose or throat. It is true we can have, according to the medical authorities, an extension of catarrhal inflammation from the nose, throat, or even from the stomach, to the mouth, and we then have acute or chronic oral catarrh, or catarrhal stomatitis; but in such cases the process is a general one, and affects not only the mucous membrane of the gums, but also that of the lips, cheeks, tongue, etc., which condition we do not have in pyorrhœa alveolaris. Dr. Patterson states that both nasal catarrh and pyorrhœa alveolaris are of an infectious nature, and further states that text-books all agree that nasal catarrh is not only contagious, but sometimes epidemic.

No less an authority than Niemeyer, in his text-book on Practical Medicine, says: "The somewhat common opinion that a cold in the head is contagious is contradicted by the experiments of Friedrich, who could not succeed in implanting the disease upon the mucous membranes of healthy persons by transferring to them secretions of persons suffering from catarrh in its several stages." Regarding the infectious nature of nasal catarrh, the same author (Niemeyer) says that the epidemic form of coryza is probably only a symptom of constitutional disease. So far as I know, Ziemssen is the only authority who favors the theory of the contagious nature of coryza. All the other authors whose writings I have consulted (including the works of Quain, Pepper, Reynolds, Niemeyer, Bosworth and others), either ignore or deny the contagious theory of this disease, or else speak of it as doubtful. Even Ziemssen admits that, up to the present time, no one has succeeded in demonstrating, by experiment, the contagiousness of coryza, and that all such attempts have resulted negatively. Dr. Patterson states that the contagious nature of pyorrhœa alveolaris is well known to careful observers. The only argument he presents in favor of its contagious nature is that the disease exhibits a tendency to spread from one or more teeth to the adjoining ones, until all are affected.

I think anyone will admit that such a statement is far from proving conclusively that the disease is contagious, in the sense generally implied by that term, although I notice Ziemssen uses a similar argument in favor of the contagious nature of coryza. The disease does sometimes show a tendency to spread in this manner, but not always; and again, in cases in which the inflammation is general and severe, it may spread to the mucous membrane of the fauces and pharynx. But are we to infer from this that the disease is contagious? We sometimes have a similar extension of the inflammatory process in cases of salivary deposit (on the molars especially), without any indication of real pyorrhœa alveolaris; and yet I do not think anyone will claim that the dyscrasia causing salivary deposit is capable of being propagated from one person to another in any other manner than by hereditary transmission. To my mind, pyorrhœa alveolaris is certainly not contagious, if we use that term in the sense generally implied. If it were so, we would expect dentists to be the principal sufferers, but we know they are not. There may be some instances in which the disease appears to be infectious. The epidemic said to have occurred in St. Gall, Switzerland, in 1876, if the reports be authentic, would be an instance of this kind. In this epidemic the disease was said to be very severe, and investigation demonstrated the presence of numerous parasites (leptothrix, bacteria, &c.) in the secreted matter, but no pus corpuscles. Schlenker, who studied these cases, concluded that the presence of the parasites was the cause of the inflammation of the root membrane. Some observers, among them being Dr. G. V. Black of Illinois, and Dr. Witzel of Germany, believe that the disease is caused by a certain species of fungus. We cannot deny the possibility of such a mode of origin, although I think no one has yet been able to demonstrate, by actual experiment, that there is any specific virus or contagium in this disease. I have endeavored to transmit the disease by inoculation from the human subject to the dog, but so far have been unable to produce anything except a negative result. My method has been to make a slight incision between the gum and the prominent cuspid tooth of the dog, and inoculate with the fresh discharge carried on an instrument directly from the patient to the animal in the next room. It may be that the lower animals are not capable of developing the disease. How that is, I do not know; but I have not, for very manifest reasons, attempted to experiment in this direction upon the human subject. It might be interesting and instructive to experiment in the mouth of a patient affected with this disease, by inoculating from the socket of an affected tooth to one not affected (by applying the discharge to a denuded surface), and observing the result. I have not as yet experimented in this manner to any great extent. Dr. Patterson states that there is the same tendency to destruction of periosteum and underlying bone in this disease as in nasal catarrh. I beg to differ with him in regard to the tendency to destruction of bone in pyorrhœa alveolaris. In nasal catarrh, when the bone is involved the process is one of caries, or necrosis, while in pyorrhœa alveolaris I think it is only very rarely that we have such a condition; but of this we shall speak further in treating of the pathology of the disease.

Salivary, serumal, or sanguinary calcular deposits, are by many believed to be the most frequent cause of pyorrhœa alveolaris. Some say it is only the sanguinary or serumal variety that is the direct cause of the symptoms of this disease. I believe that in most cases salivary calculus is the exciting cause of the sanguinary, and that after the disease is instituted the salivary assists in forming the deposits on the roots. The local irritation caused by foreign bodies in the alveoli, such as tooth-brush bristles, fish bones, splinters of wood, tooth picks, or parts of fractured teeth or bones, misplaced wedges or ligatures, etc., may result in what we may call traumatic, or acute pyorrhœa alveolaris. The irritation of clasps or partial plates may also be an exciting cause of pyorrhœa. Whether or not there is always a constitutional predisposition in cases of this disease, is still a matter of much controversy. So far as my reading goes, the preponderance of opinion seems to be that there is usually a constitutional predisposition. The traumatic or acute cases, without doubt, are due entirely to local causes, since almost any irritant of the kind described will induce the disease, and the removal of the cause results in a speedy cure. Cases in which salivary calculus irritates the pericementum and causes secondary sanguinary deposit might be classed as traumatic, for the exciting cause is a foreign body; but, although to a certain extent traumatic, they cannot be called acute cases, since the disease when induced by this cause generally assumes the chronic form. Perhaps this may be accounted for by the fact that the salivary deposit increases very gradually, and the irritation is less on that account. I think that even in these cases of pyorrhœa from the irritation of salivary calculus, we must admit the presence of a constitutional predisposition (I refer now to typical cases, in which we have the deposit of sanguinary calculus and the formation of ulcerating and suppurating pockets); because we know that not all cases of salivary calculus, or of pericemental irritation, are followed by this disease. In fact, it results in comparatively few instances. We often see marked cases of pyorrhœa alveolaris in which we can find little or no deposit about the necks and crowns of the teeth (although the deposit may have been present at the inception of the disease, and been subsequently removed by mechanical or chemical means); while in other cases we frequently see abundant salivary deposits about the necks of the teeth, and would naturally suppose the same or a greater amount of pericemental irritation would exist, and yet we do not have the typical manifestations of pyorrhœa.

The principal argument used by those who favor the local theory is that the disease can be cured by local treatment; but this statement is denied by very many good authorities. It has been asserted that typical cases of pyorrhœa alveolaris are never cured by local treatment alone, and that if the disease is well advanced, especially if it be hereditary or due to mercurialization, it is never permanently cured. I am not sure that we can take such a decided ground as that. In the cases due to salivary deposit, I believe the disease can be cured by the removal of the exciting cause, and by proper local treatment, and that we can prevent its recurrence, provided we can prevent the return of the exciting cause. It is difficult to draw the line between local and constitutional origin in these cases, for if the exciting cause be salivary caculus, that in itself is often dependent upon constitutional derangement for its development. The cases in which we can find no appreciable exciting cause are the ones last amenable to treatment. In such cases neither local nor constitutional treatment, used separately or in conjunction, seems to be of much avail in removing the condition. If there be a predisposing cause in these cases (as I think there is), how can we expect to treat them rationally by constitutional remedies, when we do not know what is the predisposing cause or constitutional derangement that induces the disease? Another argument which has been advanced in favor of the local theory is that the teeth most often affected are those in the neighborhood of the opening of the salivary ducts, those standing behind others in a crowded arch, rotated teeth, or those misplaced in consequence of injury or faulty articulation. I do not believe that teeth in the immediate vicinity of the salivary ducts, or rather that the surface of such teeth which are nearest to the ducts, are especially liable to this disease.

I think I have met with more cases affecting the upper than the lower anterior teeth, and more affecting the palatal than the buccal roots of superior molars. Granting that teeth inside the arch, or rotated, or misplaced teeth, are more liable to salivary deposits because of want of use or difficulty in keeping them clean, and that teeth which have fallen backward or forward in the arch have their periosteal covering subjected to greater irritation upon the side toward which they have fallen, we may pertinently ask why are not all teeth so situated, or so few of them, when incrusted with salivary calculus or subjected to the same amount of irritation, affected by this disease? To sum up, then, we regard the traumatic or acute cases as essentially local in their origin, since they are so easily induced and are so readily amenable to local treatment without showing any tendency to recurrence; but in the idiopathic or chronic cases, or those of pyorrhœa alveolaris proper, we think the causes are both predisposing and exciting, and that there is generally, at least, a constitutional predisposition rendering the disease liable to occur under local irritation, either mechanical or chemical. Salivary calculus, I think, is the most common exciting cause. The irritation of partial plates would probably come next in order of frequency. What the predisposing cause or causes may be, we are as yet not aware. Possibly the same, or a similar influence to that which causes exostosis of the cementum, may operate in determining the origin of this affection, the difference being, that in this disease the lime salts, instead of helping to form an organized tissue, are deposited in an amorphous manner. I think I have met with a greater number of cases affecting the teeth of the superior maxilla than of the inferior; but whether or not this has been the experience of other observers, I am not aware. The disease is one of adult life, and is common to both sexes. It is very rare in young persons, except when hereditary.

POSTSCRIPT.—Since preparing this lecture for publication, I have observed in the April number of this volume an article on the same subject by Dr. J. N. Farrar. Dr. Farrar calls this affection "pocket disease of the alveolus," or "loculosis alveolaris," and divides it into several stages. His views of the pathology of pyorrhœa alveolaris are very similar to my own, as will be seen when space permits of the publication of the remaining portion of this paper. I think Dr. Farrar's diagram of the condition of the parts in this disease is defective in one respect, viz.: around the molar root where the deposit is greater and the separation of the pericementum more advanced, it does not depict to a sufficient extent the amount of absorption of bone that we would have in such a case. I have never seen a case as far advanced as the diagram would indicate, in which the absorption of the margins of the bony socket was so slight.

Dr. Farrar says the cause is not "the same in kind as that which leads to exostosis or enlargement of the root." I would like to ask on what ground that assertion is based. If not the same, I think it quite possible that the cause is very similar. Is not exostosis supposed to be due to an irritation resulting in an excitation of the osteoblasts of the pericementum to a re-performance of their original function; and is it not possible that we have the same conditions in pyorrhœa alveolaris? True, the lime salts in the latter disease do not go to form part of an organized tissue, but we think the reason is obvious. Would we expect to have organization when the conditions are such that we have an open pocket with the pericementum in an ulcerating condition, and the presence of foreign materials (saliva, food, micro-organisms, etc.) to interfere with that process? I quite agree with Dr. Farrar that this disease never begins at the ends of the roots. When there is a tendency to deposit of lime salts in that locality, if there be no abscess and no extraneous matter present, the result is not sanguinary calculus, not pyorrhœa alveolaris, but exostosis.

Dr. Farrar makes the rather peculiar and ambiguous statement, that "although necrosis of the alveolar processes is rare, the death and degeneracy of that portion of the cementum constituting one of the walls of the pocket is not common." We presume it should read "not uncommon." I hardly think the doctor is correct, if he means to state that death of a portion of the pericementum is not uncommon as a result of this disease. I presume I have not had as much experience with these cases as Dr. Farrar has had, but I have seen quite a number of them, and yet cannot recall a single instance in which there was death of a portion of the cementum while the root contained a living pulp.

Dr. Farrar says, "When in order to be explicit, it is desirable to express the recurrent form of this disease (after once cured), which is liable in cases where death or low vitality of the cemental wall of the pocket prevents reunion with the pericemental wall, the termination *osis* may be exchanged for *itis*, &c. How can such cases be called recurrent when they are really never cured? Since the disease is one characterized by the formation of pockets, if a part of the cementum dies or degenerates and thus prevents union with the pericementum, then the pockets are not and can not be obliterated, and the disease is not cured. Hence we think it is obviously incorrect to call these cases *recurrent* ones. With the other views of Dr. Farrar, as expressed in his article, I am fully in accord, and would be pleased to hear further from him upon the subject.

